# Clinical Trial Design for Testing the Stem Cell Model for the Prevention and Treatment of Cancer

**DOI:** 10.3390/cancers3022696

**Published:** 2011-06-20

**Authors:** Rishindra M. Reddy, Madhuri Kakarala, Max S. Wicha

**Affiliations:** 1 Medical Center, University of Michigan, 1500 E. Medical Center Drive, 2120 Taubman Center, Ann Arbor, MI 48109, USA; 2 Comprehensive Cancer Center, University of Michigan, 1500 E. Medical Center Drive, Ann Arbor, MI 48109, USA; E-Mails: mkakaral@med.umich.edu (M.K.); mwicha@med.umich.edu (M.S.W.)

**Keywords:** cancer stem cell model, clinical trial design, preventative therapy

## Abstract

The cancer stem cell model introduces new strategies for the prevention and treatment of cancers. In cancers that appear to follow the stem cell model, pathways such as Wnt, Notch and Hedgehog may be targeted with natural compounds such as curcumin or drugs to reduce the risk of initiation of new tumors. Disease progression of established tumors could also potentially be inhibited by targeting the tumorigenic stem cells alone, rather than aiming to reduce overall tumor size. These new approaches mandate a change in the design of clinical trials and biomarkers chosen for efficacy assessment for preventative, neoadjuvant, adjuvant, and palliative treatments. Cancer treatments could be evaluated by assessing stem cell markers before and after treatment. Targeted stem cell specific treatment of cancers may not result in “complete” or “partial” responses radiologically, as stem cell targeting may not reduce the tumor bulk, but eliminate further tumorigenic potential. These changes are discussed using breast, pancreatic, and lung cancer as examples.

## 1. Introduction

The current stochastic model for cancer initiation and progression does not adequately address the chemoresistance, radioresistance, and high recurrence rates seen in many solid organ and hematopoetic cancers. The cancer stem cell (CSC) model has been proposed as an alternate hypothesis for carcinogenesis that may explain why many different tumors have proven difficult to treat with conventional therapies. CSCs, which comprise a small subset of the tumor bulk, have the ability to self-renew indefinitely, are able to be quiescent for long periods of time, and have been shown to be resistant to chemotherapy and radiation therapy [1]. These CSC properties may contribute to a tumor's ability to persist despite treatment, and explain the potential for late recurrences in patients with long disease-free intervals. One challenge has been to identify stem cell markers that can be used to easily identify and isolate CSCs. Current methods utilize cell surface protein expression (CD24, CD44, CD133) or functional assays such as aldehyde dehydrogenase activity or Hoescht dye efflux [2]. As cancer stem cells are being identified in numerous histologies, such as hematologic malignancies, breast cancer, pancreatic cancer, and central nervous system tumors [3-6], many different regulators of stem cell survival have been discovered, including the Notch, Wnt, and Hedgehog pathways [7,8]. These pathways offer novel targets for the development of CSC specific preventative and therapeutic drug treatments. Targeted therapies for stem cells offer a host of challenges for drug clinical trial design. Traditional Phase I dose escalation studies that measure maximum drug toxicity may not be applicable to the evaluation of targeted therapies, which may not have the systemic toxicities seen with traditional therapies and may exert a maximum biological effect at the these toxic levels. Phase II studies evaluating for tumor size reduction would not be useful, as treatment of the cancer stem cell subset of the tumor may not result in whole tumor size reduction by traditional RECIST criteria. There are numerous lessons that have been learned over the last few years from clinical trials for therapies targeting specific pathways such as EGFR [9] and for cancer immunotherapies [10,11]. Using these lessons, we can better design trials for future cancer stem cell therapies.

Clinical trials for chemoprevention or cancer risk reduction (CRR) have focused upon high incidence cancers, such as breast cancer, for which there are well validated risk prediction criteria. For example, the risk for developing breast cancer can be estimated using mathematical models such as the Gail model, identifying the presence of pre-invasive lesions such as ductal carcinoma *in situ*, or detecting genetic conditions such as BRCA1 or BRCA2 mutations [12]. Cancer risk reduction trials have also targeted low incidence but highly lethal cancers, such as pancreatic cancer, for which there may be substantial benefit of improved survival by risk reduction or early diagnosis at a surgically resectable stage [13,14]. It is well known that chronic pancreatitis, identified by elevated serum amylase and lipase, CT scan changes in the pancreas, and symptomatic abdominal pain increases risk of pancreatic cancer [15]. Lung cancer risk reduction is complex. While smoking and the presence of chronic obstructive pulmonary disease (COPD) increases the risk for lung cancers, not all smokers or COPD patients are at risk for lung cancer [15,16]. We presently lack good risk prediction biomarkers or models to determine which patients are at most risk of developing lung cancer. Trials that have targeted all smokers with non specific cancer risk reductive interventions such as vitamin supplements have not only been found to lack risk reduction, but in some cases such as the CARET trial, have been stopped early as the intervention appeared to increase the risk of lung cancer [17]. Therefore, one critical factor in clinical cancer risk reduction trials is how to identify individuals at specific risk for developing cancer to enroll in such trials.

To date, natural products with pleotrophic mechanisms of activity, such as nutritional polyphenol isolates including curcumin from the spice turmeric or epigallo catechin gallate from green tea have been examined for CRR efficacy in early phase clinical trials [18,19]. Similarly, drugs working on hormonal or other pathway to target all cells, e.g., Tamoxifen or finretinide, have been tested in clinical cancer risk reduction trials [20,21]. Only a few such agents have actually been observed to reduce the risk of cancer, including tamoxifen, raloxifene, non-steroidal anti-inflammatory drugs such as celecoxib or aspirin. However, these drugs have such unacceptable potential toxicities, including venous thrombo emboli, risk of uterine cancer or heart disease, and gastrointestinal bleeding, that acceptance among eligible patients has been very poor [22,23]. Based on the stem cell model of carcinogenesis, only stem/early progenitor cells have the capacity for tumorigenesis and metastasis. Therefore it stands to reason that these stem/progenitor cells are the cells to target specifically with risk reductive interventions [24]. This presents the opportunity to investigate specific stem cell targeted risk reductive agents for cancer risk reduction efficacy with less potential for generalized toxicity [25]. Agents which have potential promise for stem cell targeted cancer risk reduction include signaling pathway inhibitors with low toxicity, such as Notch inhibitors, natural compounds such as curcumin or sulfurophane, and metabolic agents such as metformin [26-28]. We will discuss strategies for clinical drug and CRR trial design using breast, pancreatic, and lung cancers as examples.

## 2. Overview of Clinical Trial Design for Cancer Stem Cell Targeted Treatments or Cancer Risk Reduction

### 2.1. Sampling Methods and Biomarkers

One of the key aspects for evaluating drugs that target stem cells in solid organ cancers is the measurement of the percentage of stem cells within primary or metastatic tumors. The current limitation of Fine Needle Aspirations (FNAs) of tumors is that insufficient tissue may be aspirated to identify rare populations of cancer stem cells. Core needle biopsies utilize larger needles and are able to give more structural detail on the pathologic evaluation, but also may be limited by not acquiring sufficient tissue. Future work will involve evaluating peripheral blood samples for circulating cancer stem cells or circulating cytokines or proteins specific to stem cells from different malignancies. When comparing organ systems, primary breast tumors are the easiest to biopsy, and they may be accessed by palpation or by ultrasound guidance. The same tumor can be biopsied pre and post treatment in neoadjuvant trials with relatively low risk of complications [29,30]. Pancreatic cancers require a more invasive approach, with endoscopic retrograde cholangiopancreatography (ERCP) often required for the initial tumor biopsy and for later samples. ERCP has a reported complication rate as high as 1% [31], with pancreatitis and abdominal pain being the most common potential complications. Lung cancers may be diagnosed by a bronchoscopy with a biopsy of visualized endoluminal tumors, a needle biopsy by Endobronchial Ultrasound (EBUS) or blind Wang needle biopsy. Lesions that are not accessible by bronchoscopy must be biopsied by radiologic guidance, usually by computerized tomography (CT). Complications from each of these procedures include bleeding and pneumothorax, which may require chest tube placement. Reported complication rates can be as high as 38% [32]. These tumors represent the spectrum of problems associated with biopsy related assessments. All yield small amounts of tissue that may not have adequate cellularity or tumor volume to determine the effect of a chemotherapeutic agent on the stem cell percentages within a tumor. Also, repeat biopsies of the same location may result in hemorrhage, confounding the results of future biopsies from the same location.

Patients with metastatic tumors that are located in areas accessible to biopsy, such as skin lesions, may be the best candidates to enroll in clinical studies, but these patients are rare, and usually do not represent the average patient. The most common sites for metastatic disease for breast cancers are to the bone, lung, and liver, while pancreatic cancers spread locally, and then to the liver, and lung . Lung cancers can spread to the bones and brain, among other locations [33]. Each of these locations carry its own inherent difficulties with biopsies, although liver biopsies can be performed with CT or ultrasound guidance with minimal morbidity and are fairly well tolerated by patients [34]. Unfortunately, the heterogeneity of primary tumor location and of metastatic spread limits the standardization of biopsy methods within each histology. Stem cells, or their markers, must be able to be assessed from only a few hundred cells on standard FNA's. If the stem cell population is as small as 1% of the total cells, then the beginning number of stem cells from 300 cancer cells could be as few as 2–3 cells, prior to treatment. This provides a poor option for measuring the effects of a stem cell specific therapy. Alternative methods for measuring the effects include measuring the pathway specific effects of the therapy on all of the sampled cells. As mentioned, the Notch pathway is felt to be a key stem cell pathway [35] and is being evaluated as a target for therapeutic and preventative trials. Notch inhibition may be measured in the biopsy samples and prove to be a surrogate for stem cell inhibition. This would need to be validated though in numerous tumors prior to acceptance as a valid biomarker for use in clinical trials.

### 2.2. Phase I Trials

Phase I trials for cytotoxic drugs have historically been designed to identify the maximum tolerated dose (MTD) and the dose-limiting toxicity (DLT) for each therapy [36,37]. Phase 1 trials are designed to be completed within a year [37] using relatively few patients. These Phase 1 trial patients have metastatic disease and have exhausted conventional therapeutic options. Phase I trials start at a “safe” dose, based on the dosing used in pre-clinical animal models. Initial doses are often 0.1 of the MELD10 (mouse equivalent lethal dose 10), which is the dose at which 10% of mice die from drug treatment. Dose escalation from that starting point has been based on modified Fibonacci sequences with increasing doses in cohorts of 3 patients [36]. DLT has been felt to represent the dose for maximum effect, as the higher the dose, the more likely it is to work [36]. The higher the dose of drug in general the more toxic it is, resulting in side effects in normal tissue, but with more toxicity to the tumor as well [37]. This maximum dose then provides a starting point for Phase II trials.

Unfortunately, oncology drugs overall have a high failure rate in Phase I and Phase II trials [38]. Phase I trials have changed recently, as more are using biomarkers as endpoints, as opposed to toxicity data [38]. Targeted therapies are less toxic than classical cytotoxic agents, and the optimal biologic dose (OBD) may not be the same as the MTD, as presumed in earlier studies [9]. Because of less toxicity, targeted drugs may be investigated in healthy volunteers, rather than patients with end stage disease. Even in the healthy population, there is a large variation in pharmacokinetics, and thus pharmacodynamic endpoints may be valuable, even in Phase I trials [9]. In this healthy population, normal tissues may be sampled to assess the effect of a given therapy, such as blood samples or skin tissue [38]. These biopsies of normal tissue from healthy volunteers may allow one to measure a therapy's effect on normal stem cells that may be found in hematopoietic cells or in normal skin tissue [36]. Defining and validating the optimal biologic dose is another challenge in all targeted therapies [39].

Given these concerns, Phase I trials for stem cell therapeutics will likely need to start at a dose defined in pre-clinical animal models, but one that shows a demonstrable effect on either the relative stem cell population, or a defined stem cell pathway. Dose escalation may then proceed, but pharmacodynamic measurements must be performed to assess for the therapies effects, either on tumors, or in normal tissues. CSC viability may also be measured from tumor samples, but methods must first be established in early passage xenografts to allow for a quick assessment of the cancer stem cell population [40]. Endpoints for targeted therapy Phase I trials shift from MTD and DLT, to establishing the minimum target inhibiting dose (MTID) [37]. This depends on what's being measured, either tumor or normal tissue, and then if a pathway is being evaluated, or a stem cell population. Either way, it is unclear if this will ultimately translate to a survival benefit, as tumor response rates in cytotoxic agents often do not correlate with patient survival [41,42].

### 2.3. Phase II Trials

Phase II trials have been designed as single arm studies, comparing results to historical controls with the goal of identifying the most promising agents for Phase III study [43]. Unfortunately, this single arm design, which was thought to help increase the efficiency of drug development, may have actually hindered drug evaluation because of prognostic bias from older data [11,44]. As trial design has been re-evaluated, the goals of Phase II studies have also been reassessed, to include evaluating for drug activity, directing tumor selection for targeted therapies, and to serve as a gateway to Phase III trials [45]. Endpoints for cytotoxic agents have been objective tumor response, using RECIST (Response Evaluation Criteria in Solid Tumors), looking for response rates of 30%–50% reduction in tumor size. There are many limitations of using tumor size. There has been documented variability in tumor measurements by different clinicians evaluating the same CT scans [46]. Also, tumors with necrotic centers may not appear to change by RECIST criteria, although there has been a significant tumor effect [47]. While a reduction in primary tumor size may be seen with cytotoxic therapies, targeted drugs, especially ones that aim at the stem cell population, will initially only effect the small subset of tumorigenic cells, which could be as few as 1% of the tumor bulk. This makes RECIST criteria a poor choice for tracking the effects of stem cell specific therapeutics.

Alternative endpoints for Phase II trials include time to progression, progression free rate, tumor growth rate, and progression free survival rate. These may be better endpoints than tumor size, but require longer follow up to obtain clinically significant results [47]. Overall survival may be the best endpoint, but requires too long a time for follow up [45]. Biomarkers can also be used for Phase II trials, but offer significant limitations, including a lack of validation for prognostic use in newer therapies [45]. Potential markers need strong pre-clinical data that connects biomarker changes to therapeutic dosing and has a defined assay for clinical use [48]. Even if such assays are not used to evaluate target response, they may help determine if patients are eligible for certain treatments, as some tumors may not have measurable cancer stem cell numbers to allow for therapy evaluation [48]. Single-arm monotherapy trials may still have value, but randomized trials may be better to help determine optimal dosing regimens for new therapies, or to compare them to standard treatments. Blinded trials would be optimal [45].

### 2.4. Phase III Trials

Phase III trials were usually designed to enroll hundreds to thousands of patients, and randomized the new therapy to the gold standard treatment, with the goal of measuring drug efficacy. These trials have had an extremely high failure rate, with only 50% of drugs succeeding as of 2006. This failure rate may be even higher in oncology therapies. Many targeted therapies have failed in Phase III because of concerns ranging from toxicity to efficacy issues [49]. Other methods of failure in immunotherapy trials have included lack of time for follow up, as cytotoxic agents have historically shown an effect earlier prompting shorter follow-up times in the designs of the trials, and the high costs of running Phase III trials, which can limit even “successful” drugs from being fully evaluated, trials may be stopped prematurely if the new therapeutic appears unsuccessful early on [11]. These trials often required at least 2–5 years to complete, with extremely high costs to the pharmaceutical company supporting the research [37].

When considering CSC therapeutics, two factors must be taken into consideration: patient selection and trial design. One may target only patients with tumors which express a high cancer stem cell content based on pre-treatment evaluation. Patients with low stem cell percentages may not have a way to measure the therapeutic effect. This again raises the concerns of sampling and adequate biomarkers in these trials [37]. The second consideration is in designing appropriate end points to measure the drug's efficacy on stem cells, whether overall tumor growth or patient survival. These two concerns are critical. A meaningful response can be missed if too many patients with tumors have low stem cell percentages, and an effect is not detected by measuring stem cell reduction. This would appear to indicate that the tumors are not sensitive to stem cell therapies, but it may be that the tumors were not the right model to evaluate for a stem cell response [50]. Responses can also be missed because the planned end points of the trial are too short to identify the slower acting cytostatic effects of stem cell inhibition. Studies can be designed to add stem cell therapies to standard chemotherapies, which may allow one to see a quicker effect, using cytotoxic and cytostatic therapies in conjunction [9]. Overall survival is the best end point, but adds to the length of follow-up time of a trial and adds significantly to the costs. Alternative end points for Phase III trials may be similar to those for Phase II design, such as time to progression, progression free rate, and symptom palliation. Appropriately selected end points may salvage a number of therapies that could have otherwise been deemed “failures” by using traditional designs, as has been seen in immunotherapy trials [10].

## 3. Stem Cell Targeted Cancer Risk Reduction Clinical Trials

### 3.1. Sampling Methods and Biomarkers for CCR Trials

As with cancer treatment clinical trials, cancer risk reduction clinical trials are limited by the need for biomarkers to assess intervention efficacy, since a cancer incidence endpoint requires very large patient numbers and long follow-up times, both of which are prohibitively expensive for investigating new as yet unproven interventions [51]. One challenge in clinical cancer prevention has been that biomarkers used to date have been associated with risk of cancer, e.g., Mammographic breast density or inflammatory cytokines such as prostaglandin E2, but have not been integrally implicated in the carcinogenesis process, per se [52]. Therefore, there is a great need for novel biomarkers of efficacy which are integrally involved in carcinogenesis. As mentioned earlier, stem cell percentage in a given tissue or signaling pathways, such as Wnt, Notch and Hedgehog, may be attractive biomarkers for cancer risk reduction efficacy [53]. Other potential stem cell specific biomarkers include expression of the polycomb cassette of proteins BMI1, EZH2 or SUZ12 expressed in breast tissue [54]. These polycomb proteins regulate whether a stem cell self renews or differentiates. Cancer risk reduction clinical trials also are limited in the means by which they can access tissue for biomarker assessment. Although patients with existing cancer may find a 1% risk of developing a complication from a biopsy procedure such as ERCP for pancreas acceptable, the threshold for acceptable risk is much more stringent for cancer risk reduction trials in which the participants are at risk but do not already have disease. Core biopsy of the breast before and after a risk reductive intervention, may cause emotional distress, bleeding and scarring which could limit trial approval through institutional review boards or trial accrual [55]. Similarly, bronchoscopic lung biopsy with risk of bleeding or even pneumothorax or ERCP for pancreas biopsy with risk of abdominal pain and infection are problematic in cancer risk reduction clinical trials [56]. Such approaches may also not yield sufficient tissue to isolate the relative rare stem cell populations and determine meaningful differences in the stem cell percentage or signaling. Other approaches such as random periareolar fine needle aspiration or ductal lavage previously used in breast cancer risk reductive trials are not applicable for use in assessing efficacy of stem cell targeted intervention trials [57].

Stem cell basic science research has yet to identify changes within stem cells which reflect an increased risk of transformation to malignancy or in other words changes which identify an “initiated stem cell”. Ideally, for cancer risk reduction one would aim to target stem cells in which there has already been some premalignant change rather than the general population of normal stem cells in a given organ system. Normal tissue specific stem cells serve a critical function of being able to participate in tissue repair and homeostasis so one would not wish to nonspecifically kill stem cells in any organ system [58]. Current approaches to stem cell targeted cancer risk reduction have focused on interventions which limit stem cell self renewal without killing the stem cell because dysregulated stem cell self renewal has been proposed to lead to clonal expansion as an early step in transformation to malignancy [25]. Some stem cell specific proteins and miRNA are currently being investigated as potential biomarkers of risk, including the polycomb protein EZH2 expression which has been shown to increase serially from normal to atypical ductal hyperplasia to ductal carcinoma i*n situ* and finally to invasive cancer [54]. Changes in such stem cell specific biomarkers of risk could also be used as biomarkers of intervention efficacy if validated against a cancer incidence or mortality outcome. To move forward in stem cell targeted cancer risk reductive intervention clinical trials, assessment of appropriateness and validity of risk and efficacy biomarkers is critical. These areas of stem cell targeting for cancer treatment or cancer risk reduction represent paradigm shifting strategies which could yield less toxic and more effective therapies if successful.

### 3.2. Clinical Trial Design for Stem Cell Targeted Cancer Risk Reductive Interventions

The trial design which is likely to yield the best opportunity to investigate the stem cell specific effects of a given intervention in Phase II or III clinical trials is a “neoadjuvant” design. In a neoadjuvant cancer treatment approach, a pre-treatment biopsy must be taken and then the intervention is typically given for 14–28 days prior to surgery in which the entire remaining tumor is removed [59]. Changes in the biomarkers selected are compared between the core biopsy pre-treatment and the larger volume of surgical tissue post treatment [60]. For cancer risk reduction trials in the breast for e.g., there are certain conditions in which risk of cancer is so high, as in BRCA1 mutation carriers who have 30%-70% lifetime risk of developing breast cancer that the standard of care is to offer risk reduction mastectomy [61]. In such a scenario, agents could be tested in randomized trial with risk reductive stem cell targeting drug or other intervention prior to scheduled surgery to allow access to sufficient tissue to isolate rare stem cell populations. High risk ductal carcinoma *in situ* by Van Nuys criteria is another such clinical condition in which surgery is indicated for invasive cancer risk reduction [62]. Severe inflammatory bowel disease (IBD) is a gastrointestinal condition in which there may be up to a 30 fold increase in risk of developing colon cancer and often a segment of the affected colon is surgically removed to decrease such risk and symptoms from IBD [63]. A neoadjuvant approach for cancer risk reduction is not as feasible for pancreatic or lung cancers. However, unlike BRCA1 or BRCA2 mutation carriers who may have field cancerization changes, in the high risk preinvasive breast conditions such as ductal carcinoma *in situ*, one must assess concordance between pretreatment core biopsy generally taken from within the center of the lesion and the residual tissue resected in the final surgery [64]. Stem cells have been shown to reside at the leading invasive edge of breast cancers and preinvasive breast lesions such as DCIS so geographic distribution within a lesion may affect the stem cell percentage seen independent of the intervention [65]. Therefore, the inherent variability of assessing the number or percent of the rare stem cells in core biopsies versus mastectomy samples must be validated prior to the use of stem cells as biomarkers in DCIS trials. Stem cells as biomarkers of cancer risk reductive agent efficacy is perhaps more challenging than treatment trials where patients with Stage IV disease may have large volumes of tissue from which to detect stem cell changes.

## 4. Conclusions

The cancer stem cell model offers new insights into the limitations of current cancer treatments. Stem cell specific therapies offer new targets, whose treatment may or may not eradicate tumors, but should prevent them from spreading, by eliminating the malignant potential. Cancer risk reduction is an attractive option to target these stem cells, as most risk reductive agents are natural compounds or drugs with lower toxicities than standard chemotherapies. The key to evaluating these new therapies is the proper design of Phase I, II, and III clinical trials to accurately assess the effects of these treatments. Tumor response by size criteria will not reflect a stem cell specific therapy's effects. Clinical end points such as progression free rate or overall survival, while taking longer to assess and more expensive than current end points, will be the best way to assess stem cell therapies' effects.
